# The Effect of a Traditional Chinese Medicine Course on Western Medicine Students’ Attitudes Toward Traditional Chinese Medicine: Self-Controlled Pre-Post Questionnaire Study

**DOI:** 10.2196/55972

**Published:** 2026-01-16

**Authors:** Haoyu He, Hanjin Cui, Li Guo, Yanhui Liao

**Affiliations:** 1 Department of Psychology, College of Education, Hunan First Normal University, Changsha, Hunan, China; 2 Research Center for Innovative Development of Teacher Education in the New Era, Changsha, China; 3 Hunan Key Laboratory for Children's Psychological Development and Brain & Cognition Science, Changsha, China; 4 Institute of Integrative Medicine, Xiangya Hospital, Central South University, Changsha, Hunan, China; 5 Department of Educational Administration, The Second People's Hospital of Hunan Province (Brain Hospital of Hunan Province), Changsha, China; 6 Department of Psychiatry, Sir Run Run Shaw Hospital, Zhejiang University School of Medicine, 3 East Qingchun Road, Hangzhou, Zhejiang, 310016, China, 86 18814898844

**Keywords:** traditional Chinese medicine, TCM, Chinese medicine, natural medicine, naturopathy, naturopathic, Western medicine students, Western medicine, modern medicine, medical students, attitude

## Abstract

**Background:**

Traditional Chinese medicine (TCM) hasbeen widely used to treat various diseases in China for thousands of years and has shown satisfactory effectiveness. However, many surveys found that TCM receives little recognition from Western medicine (WM) physicians and students. At present, TCM is offered as a compulsory course for WM students in WM schools.

**Objective:**

This study aimed to investigate whether TCM courses can affect the WM students’ attitude toward TCM.

**Methods:**

WM students from Xiangya Medical School were invited to completeaweb-based questionnaire before and immediately after a TCM course. Their attitude toward TCM and treatment preferences for different kinds of diseases were tested. The Attitude Scale of TCM (ASTCM) was used. The main part of the ASTCM was designed to measure the attitude of medical students towardTCM. It consisted of 18 items, divided into cognitive dimension (5 terms), emotional dimension (8 terms), and behavioral tendencyfactor (5 terms).

**Results:**

Finally, the results of 118 five-year program (FYP) and 36 eight-year program (EYP) students were included. For FYP students, there was a significant increase in the total mean score (66.42, SD 7.66 vs 71.43, SD 7.38;*P*<.001) of ASTCM after the TCM course. Significant increases in mean scores of the 3 factors of attitude (cognition: 21.64, SD 2.08 vs 22.90, SD 1.94; affection: 25.21, SD 4.39 vs 27.96, SD 4.4; and behavioral tendency: 19.577, SD 3.02 vs 20.58, SD 2.76; *P*<.001)were also observed. Except for the score of behavioral tendency (17.50, SD 3.54 vs 18.78, SD 3.22; *P*=.02), a significant increase was not detected in total score, cognition, and affection in EPY students (total score: mean 60.36, SD 10.53 vs mean 62.92, SD 10.05; cognition: mean 20.50, SD 2.73 vs mean 20.69, SD 2.73; and affection: mean 22.36, SD 6.32 vs mean 23.44, SD 5.84; all *P*>.05). The treatment preference of FYP students in acute (*P*=.02), chronic (*P*=.003), and physical diseases (*P*=.02) showed remarkable change. A major change was also detected in internal diseases (*P*=.02), surgical diseases (perioperative period; *P*=.01), and mental illnesses (*P*=.02) in EYP students. This change mainly appeared as a decline in WM preference and an increase in TCM and WM preference.

**Conclusions:**

The study showed that earlier exposure to the TCM course increased the positive attitude toward TCM in students majoring in WM. The results provide some suggestions for arraging TCM courses in WM schools.

## Introduction

Traditional Chinese medicine (TCM) has been widely used to treat various diseases in China for more than 2500 years, and it has become an integral part of Chinese culture [1]. On the basis of its holistic theory and rich practical experiences, TCM offers a diversity of therapeutic strategies for complex diseases to balance the Yin and Yang disorders of the human body [2,3].

However, Western medicine (WM) has become the mainstream medicine in modern China [4]. The number of TCM hospitals is only approximately one-fifth of the number of WM hospitals, with the number of TCM practitioners less than a quarter of that of WM physicians [5]. Medical colleges mainly focus on WM teaching, except for several colleges of TCM [6]. The curricula that WM students learn include basic and clinical medical courses, which are similar to European and American programs. Basic medical courses include human anatomy, embryology, biochemistry, physiology, pathology, pharmacology, and so on. These courses provide a theoretical foundation for clinical medicine. Clinical medicine courses focus more on practice, including internal medicine, surgery, obstetrics and gynecology, pediatrics, neurology, infectious diseases, and so on. These courses help studentsmaster the diagnostic and treatment skills needed for managing diseases. Thus, TCM receives less recognition from WM physicians and students [4]. A study among Hong Kong WM physicians showed that only 11.9% and 9.4% of them had ever considered or referred their patients to TCM, respectively [7]. Another survey found that medical students appeared to become more negative toward TCM after learning WM [8]. In addition, the positive attitude held by WM students toward TCM was less than half reported by some other surveys [8-10]. The present situation due to the negative attitude of WM students toward TCM greatly hinders the inheritance, development, and innovation of TCM in China [11]. Attitude refers to a constant psychological inclination that individuals hold toward other individuals, ideas, emotions, or events. It encompasses a subjective evaluation and influences the behavior of individuals [12]. Cognition, affection, and behavioral tendency are the 3 critical components of attitude. Among them, the cognitive component is the foundation of the other components of attitude. The emotional component is the core and key of attitude, which affects both cognitive and behavioral components. Behavioral tendency components can affect people’s future reactions to attitude objects. The 3 components are interrelated and mutually restrictive [13]. Consequently, cultivating a suitable attitude toward TCM will serve to foster a precise perspective on TCM for WM students.

TCM courses in WM schools are a direct way to rectify the attitude. A survey conducted at the Capital Medical University suggested that after finishing the TCM course, more than 65% and 71.4% of WM students approved the safety and efficacy of TCM, respectively; 94.3% of students believed that TCM was worth learning; and 82.9% of students found that TCM was helpful for clinical practice [14]. It is believed that the course lets WM students understand the basic ideas, theories, and skills of TCM. Meanwhile, the course helps them understand the differences between TCM and WM, broadens their mind, and enriches the strategies of diagnosis and treatment. Students should possess a profound understanding of both TCM and WM and offer better medical service for patients in their future clinical practice [14,15].

Xiangya School of Medicine was founded in 1914 by the Hunan Society of Education and the Yale Association. It was the first WM school in China. At present, at Xiangya School of Medicine of Central South University, TCM is offered as a compulsory course for grade 3 WM students in the 5-year program (FYP) and grade 5 in the 8-year program (EYP). It was the first time for both of them to access the TCM course. TCM is, however, less recognized by WM physicians and students, posing challenges for its integration into modern medical education as a compulsory course. This study used a self-controlled design with online questionnaires administered before and after the TCM course to WM students. The aim of the study was to explore whether TCM courses can exert an impact on the attitude of WM students toward TCM. The a priori hypothesis is that the TCM course will significantly improve WM students’ attitudes toward TCM, particularly among FYP students, because of their relatively less established WM knowledge base compared to EYP students. The results are most likely to be used by medical educators, medical education researchers, WM students, future clinicians, and health care policy makers.

## Methods

### Description of TCM Course

According to the teaching arrangement of the dean’s office at Xiangya Hospital, the TCM course for FYP and EYP students was taught by the same teaching group in the second semester of 2022—from April 15, 2022, to June 10, 2022, once a week for students in FYP, and from June 21, 2022, to August 15, 2022, once a week for students in EYP. Each theoretical part (20 class h in total) was followed by an internship (20 class h in total). The course includes the basic theory of TCM, diagnostics of TCM, Chinese pharmacy, Chinese medical formulas, acupuncture, and moxibustion. All the students were required to study *Approaching TCM,* which is a synchronous online course recorded by teachers from the Department of TCM in Xiangya Hospital. In addition, they were required to finish the self-examination after each chapter. The TCM course was delivered by teachers from the department of TCM in Xiangya Hospital. All the teachers are clinical physicians with medium or senior titles, holding qualification certificates for physicians and teacher qualifications from colleges and universities. *Traditional Chinese Medicine, Ninth Edition*, published by the People’s Health Publishing House, was used as the textbook. According to the syllabus, the basic theory of TCM, diagnostics of TCM, traditional Chinese pharmacology, science of prescriptions, and acupuncture and moxibustion were taught.

### Participants and Procedure

The participants comprised 145 grade 3 students in an FYP and 37 grade 5 students in an EYP. The students were asked to complete the same questionnaire before and after the TCM course online.

### Ethical Considerations

This study was approved by the Ethics Committee of Hunan First Normal University (202202; Multimedia Appendix 1). Informed consent was obtained from all participants by choosing “yes” at the beginning of the online questionnaire. The information provided by participants will not be disclosed to any third party and will be used solely for the purposes of this research. All participant identifiers have been anonymized to ensure confidentiality. As the intervention involved regular academic coursework during a natural semester and posed no risk of harm, no compensation was provided to participants on ethical grounds.

### Measures

The questionnaire comprised four parts. The first part was the informed consent, including the purpose and risk of the study, the benefits of participation, and confidentiality. Part two was the basic demographic information(eg, name, sex, age, grade, and educational program). Part three was the Attitude Scale of TCM (ASTCM) developed by Wu [16]. This scale consists of 18 items divided into cognitive dimension (5 terms), emotional dimension (8 terms), and behavioral tendency factor (5 terms). The reliability test showed that internal consistency coefficients (ranging from 0.78 to 0.85), split-half reliability (ranging from 0.64 to 0.84), and retest reliability (ranging from 0.62 to 0.85) were significant. The validity test showed that the explanatory rate of the 3 factors from exploratory factor analysis was 53.58%. Confirmatory factor analysis showed that the root mean square error of approximation, comparative fit index, and non-normed fit index were 0.067, 0.903, and 0.995, respectively. The ASTCM was designed to measure the attitude of medical students toward TCM, and the reliability and validity indicators meet the measurement requirements. Physical illness is the opposite of “mental illness.” There are pathological changes in the structure of body tissues (organs) or disruptions in physiological functions. Part 4 was set to assess treatmentpreferences for different kinds of diseases [17], for example, whether participants would choose TCM, WM, or a combination of the two to treat functional disorders.

### Statistical Analysis

Analyses were carried out using SPSS (version 23; IBM Corp). For data with homogeneous variance, the intergroup differences in attitude scores (grouped by demographic information) were analyzed using an independent sample 2-tailed *t* test, and intragroup differences (self-controlled) were analyzed using a paired 2-tailed *t* test. For data with heterogeneous variance, the intergroup differences in attitude scores (grouped by demographic information) were analyzed using the Mann-Whitney *U* test, and the intragroup differences (self-controlled) were analyzed using the Wilcoxon *W* test.

Paired sample 2-tailed *t* tests were used to analyze changes in the total, cognition, affection, and behavioral tendency scores between the pretest and posttest. The McNemar test was used for analyzing changes in the selection preference of different therapeutic strategies. Cohen *d* was used to measure the effect sizes of changes from pre- to postcourse using the following formula: $d=\frac{t_{matched}}{\sqrt{\frac{n}{2}}\sqrt{\frac{1}{1-\rho }}}$,where *n* is the paired sample size and *ρ* refers to the correlation coefficient. Cohen suggested that *d*=0.2 be considered a “small” effect size, 0.5 represents a “medium” effect size, and 0.8 represents a “large” effect size [18].

## Results

The data were collected from April 15, 2022, to August 15, 2022. In total, 118 FYP and 36 EYP students completed the before and after tests, and the data analysis was completed.

### Basic Demographic Information of Participants

There were 118 and 36 questionnaires returned from 145 students in the FYP and 37 students in the EYP, respectively. Among the returned questionnaires, 118 (FYP) and 36 (EYP) were valid with before and after tests completed. Finally, 118 (FYP) and 36 (EYP) questionnaires were included for statistical analysis.

The basic demographic information of participants is presented in Table 1. The sample was 39.8% (n=47) male and 60.2% (n=71) female participants in FYP, and 38.9% (n=14) male and 61.1% (n=22) female participants in EYP(question 6 of the questionnaire). The average age was 20.64 (SD 0.70) years and 22.56 (SD 0.56) years for FYP and EYP, respectively. Of 118 FYP students, 18 (15.3%) reported that they had relatives engaged in TCM clinical or scientific research (question 9), whereas this percentage was 16.7% (6/36) among EYP students. Moreover, 74.6% (88/118) of FYP students and 66.7% (24/136) of EYP students reported that they had received TCM treatment (question 10). In addition, 64.4% (76/118) of FYP students and 52.8% (19/36) of EYP students reported that there were celebrities in the field of TCM whom they admired (question 11; Table 1).

**Table 1.** Basic demographic information of participants.

Characteristics	FYP^a^students (n=118)	EYP^b^ students (n=36)
Sex, n (%)		
Male	47 (39.8)	14 (38.9)
Female	71 (60.2)	22 (61.1)
Age (y), mean (SD)	20.64 (0.70)	22.56 (0.56)
Students whose relatives engaged in TCM^c^ clinical or scientific research, n (%)	18 (15.3)	6 (16.7)
Students who had received TCM treatment, n (%)	88 (74.6)	24 (66.7)
Students who admired celebrities in the field of TCM, n (%)	76 (64.4)	19 (52.8)

a FYP: 5-year program.

b EYP: 8-year program.

c TCM: traditional Chinese medicine.

There was no significant difference in the attitude pretest scores of FYP students on questions 5, 9, and 10, but there was a significant difference on question 11. On question 11, the total attitude score and 3-factor scores of students who answered yes were significantly higher than those who answered no (Table 2). We did not find significant differences in the pretest scores and 3-factor scores of EYP students in terms of demographic factors.

**Table 2.** The difference in the attitude pretest scores of 5-year program students (n=118) on question 11.

Score	Do you have any celebrities in the field of TCM^a^ whom you admire?		*t* test^b^ (*df*)	*P* value	Cohen *d*
	Yes (n=76), mean (SD)	No (n=42), mean (SD)			
Total score	68.26 (7.15)	63.07 (7.50)	3.711 (116)	<.001	0.71
Cognition score	21.97 (2.03)	21.02 (2.07)	2.421 (116)	.02	0.46
Affection score	25.95 (4.19)	23.88 (4.46)	2.506 (116)	.01	0.48
Behavior tendency score	20.34 (3.04)	18.17 (2.76)	3.848 (116)	<.001	0.74

a TCM: traditional Chinese medicine.

b Independent sample 2-tailed *t* test.

### The Effect of TCM Course on Attitude Toward TCM

For FYP students, there was a significant increase in the total mean score of ASTCM after the TCM course (from 66.42, SD 7.66 to 71.43, SD 7.38; *P*<.001). A significant increase was also observed in the mean scores of the 3 factors: cognition (from 21.64, SD 2.08 to 22.90, SD 1.94; *P*<.001), affection (from 25.21, SD 4.39to 27.96, SD 4.4; *P*<.001), and behavioral tendency (from 19.57, SD 3.02 to 20.58, SD 2.76*; P*<.001). Interestingly, except for the behavioral tendency mean score (from 17.50, SD 3.54 to 18.78, SD 3.22; *P*=.02), a significant increase was not detected in the total mean score (from 60.36, SD 10.53 to 62.92, SD 10.05; *P*=.19), cognition (from 20.50, SD 2.73 to 20.69, SD 2.73; *P*=.69), and affection of ASTCM (from 22.36, SD 6.32 to 23.44, SD 5.84; *P*=.34) in EPY students (Table 3). The effect sizes (Cohen *d*) are presented in Table 3.

**Table 3.** Changes in attitude scores of Western medicine students.

Score	FYP^a^ (n=118)					EYP^b^ (n=36)				
	Precourse, mean (SD)	Postcourse, mean (SD)	*t* test^c^ (*df*)	*P* value	Cohen *d*	Precourse, mean (SD)	Postcourse, mean (SD)	*t* test (*df*)	*P* value	Cohen *d*
Total score	66.42 (7.66)	71.43 (7.38)	−7.206 (117)	<.001	−0.67	60.36 (10.53)	62.92 (10.05)	−1.353 (35)	.19	−0.25
Cognition score	21.64 (2.08)	22.90 (1.94)	−6.276 (117)	<.001	−0.63	20.50 (2.73)	20.69 (2.73)	−0.397 (35)	.69	−0.07
Affection score	25.21 (4.39)	27.96 (4.45)	−6.912 (117)	<.001	−0.6	22.36 (6.32)	23.44 (5.84)	−0.976 (35)	.34	−0.18
Behavioral tendency score	19.57 (3.02)	20.58 (2.76)	−3.892 (117)	<.001	−0.34	17.50 (3.54)	18.78 (3.22)	−2.354 (35)	.02	−0.38

a FYP: 5-year program.

b EYP: 8-year program.

c Paired sample 2-tailed *t* test.

In addition, we found that the attitude scores of FYP students significantly increased after the TCM course, regardless of whether they admired a Chinese medicine celebrity. However, no significant difference was observed between students who admired a TCM celebrity and those who did not in the posttest attitude scores(Table 4).

**Table 4.** The difference in the attitude scores between pretest and posttest of 5-year program students on question 11 (n=118).

Score	Do you have any celebrities in the field of TCM^a^ whom you admire?									
	Yes (n=76)					No (n=42)				
	Precourse, mean (SD)	Postcourse, mean (SD)^b^	*t* test^c^ (*df*)	*P* value	Cohen *d*	Precourse, mean (SD)	Postcourse, mean (SD)^b^	*t* test (*df*)	*P* value	Cohen *d*
Total score	68.26 (7.15)	72.54 (5.13)	−5.803 (75)	<.001	−0.60	63.07 (7.50)	69.63 (10.04)	−4.472 (41)	<.001	−0.70
Cognition score	21.97 (2.03)	23.14 (1.46)	−5.221 (75)	<.001	−0.58	21.02 (2.07)	22.45 (2.55)	−3.606 (41)	.001	−0.61
Affection score	25.95 (4.19)	28.42 (3.70)	−5.608 (75)	<.001	−0.55	23.88 (4.46)	27.12 (5.52)	−4.140 (41)	<.001	−0.64
Behavioral tendency score	20.34 (3.04)	20.97 (2.13)	−2.120 (75)	.04	−0.22	18.17 (2.76)	19.86 (3.55)	−3.548 (41)	.001	-0.53

a TCM: traditional Chinese medicine.

b Due to the heterogeneity of variance, we compared the posttest attitude scores between groups using the Mann-Whitney *U* test, and the result showed no significant difference.

c Paired sample 2-tailed *t* test.

### The Effect of TCM Course on Selection Preference of Different Therapeutic Strategies

For FYP students, there was a significant difference in the selection preferences of different therapeutic strategies for acute (*P*=.025), chronic (*P*=.02), and physical diseases (*P*=.004). After the TCM course, the percentage of students who preferred WM for acute diseases decreased from 94.1% (n=111) to 83.9% (n=99). The preference for the combination of TCM and WM (TCM and WM) increased from 5.1% (n=6) to 14.4% (n=17), and the preference for TCM increased from 0.8% (n=1) to 1.7% (n=2). For chronic diseases, fewer students preferred WM (from n=10, 8.5% to n=1, 0.8%), whereas more students preferred TCM (from n=53, 44.9% to n=72, 61%). As for physical diseases, the percentage of students preferring WM declined (from n=56, 47.5% to n=39, 33.1%), whereas the preference for TCM and WM increased (from n=52, 44.1% to n=78, 66.1%; Table 5).

For EYP students, the significant difference in the selection preferences for different therapeutic strategies mainly focused on internal diseases (*P*=.02), surgical diseases in the perioperative period (*P*=.01), and mental illnesses (*P*=.02). More students realized that TCM and WM was a better choice (from n=12, 33.3%-52.8%) for internal diseases. The percentage of students who preferred TCM and WM for perioperative surgical diseases nearly tripled from 8.3% (n=3) to 25% (n=9). There was also an approximately 20% point increase in WM and TCM preference for mental illnesses (from n=51, 41.7% to n=22, 61.1%) with a marked decline in WM (n=19, 52.8% to n=13, 36.1%; Table 3).

**Table 5.** Changein treatment preference of Western medicine (WM) students (n=118).

Program, disease, treatment preference, and data type				Before course, n (%)	After course, n (%)	*P* value^a^
FYP^b^						
Acute diseases						.02
TCM^c^				1 (0.8)	2 (1.7)	
WM				111 (94.1)	99 (83.9)	
TCM and WM				6 (5.1)	17 (14.4)	
Chronic diseases						.003
TCM				53 (44.9)	72 (61)	
WM				10 (8.5)	1 (0.8)	
TCM and WM				55 (46.6)	45 (38.1)	
Somatic disease						.02
TCM				10 (8.5)	7 (5.9)	
WM				56 (47.5)	39 (33.1)	
TCM and WM				52 (44.1)	78 (61)	
EYP^d^						
Internal diseases						.02
TCM				4 (11.1)	2 (5.6)	
WM				20 (55.6)	15 (41.7)	
TCM and WM				12 (33.3)	19 (52.8)	
Surgical diseases (perioperative period)						.01
TCM				1 (2.8)	1 (2.8)	
WM				32 (88.9)	26 (72.2)	
TCM and WM				3 (8.3)	9 (25)	
Mental illnesses						.02
TCM				2 (5.6)	1 (2.8)	
WM				19 (52.8)	13 (36.1)	
TCM and WM				15 (41.7)	22 (61.1)	

a McNemar test.

b FYP: 5-year program.

c TCM: traditional Chinese medicine.

d EYP: 8-year program.

## Discussion

### Principal Findings

This study was conducted in natural classes, based on the real teaching arrangements of the dean’s office at Xiangya Hospital. The study examined the effect of TCM courses on the attitude toward TCM in students majoring in WM using the ASTCM. The study found that the course significantly increased the total score and the scores of the 3 factors of ASTCM in FYP students but did not change these scores in EYP students, except for the score of the behavioral factor. The study also found a significant change in therapeutic strategy preferences before and after TCM learning, with the percentage reduction in WM and increase in TCM or TCM and WM. The alteration in therapeutic approach inclination primarily manifested in acute, chronic, and somatic ailments among FYP scholars and in internal, perioperative surgical, and psychological disorders in EYP learners. The results suggested that TCM courses increased a positive attitude toward TCM for WM students.

### Basic Demographic Information of Participants

The demographic results showed that there were more female students in both FYP and EYP groups. The result was similar to the gender ratio officially reported by Central South University. The age of EYP students was 2 years older than that of FYP students. This was in line with the reality. An interesting result was that FYP students who admired Chinese medicine celebrities had a significantly better attitude toward TCM than those who did not admire Chinese medicine celebrities. This, to some extent, indicates that celebrity worship had a positive impact on attitudes toward TCM [19].

### The Effect of TCM Course on Cognitive Component of Attitude in FYP

This study reported a more positive attitude toward TCM in FYP students after completing the TCM course. The results were supported by another study carried out at the Capital Medical University, which found that WM students became more positive toward TCM after learning about it [20]. Attitude is a disposition toward or against a specified phenomenon, person, or thing. An attitude comprises cognitive, affective, and behavioral tendency components [21]. It is believed that attitude canbe changed by interventions or messages delivered at a particular time from the perspective of cognition, affection, and behavioral tendency [22].

TCM is generally considered to be mysterious, profound, and lacking a scientific basis [23]. By learning the basic theory of TCM, students gained a preliminary understanding of the basic theoretical framework of TCM. They learned that TCM is not mystical but has a solid foundation to support. They also learned how TCM physicians diagnose diseases with their unique syndrome differentiation system by learning the diagnostics of TCM. They witnessed how to use so-called “flowers and plants” (herbs) to treat diseases by learning Chinese pharmacy and medical formulas. In the acupuncture and moxibustion courses, the mysterious meridians were demystified and presented to students. All the abovementioned courses are believed to improve the students’ attitude toward TCM from the perspective of cognition.

### The Effect of TCM Course on Affective Component of Attitude in FYP

Improvement in the affection component of attitude was also observed after the TCM course. It is a form of identification and resonance. First, TCM was acknowledged by students rationally with correct cognition via theoretical lectures. Second, identification and resonance of students were achieved through internships, which are considered the most crucial experiences for undergraduate medical students [24]. The students witnessed the remarkable therapeutic effect of TCM. The cases showed that 2 doses of medicine cured a 3-month persistent cough, and immediate acupuncture for acute knee injuries were often shown. Some students even experienced TCM for dysmenorrhea: the entire diagnosis and treatment process, from pulse feeling to prescription. Finally, during the study, many famous TCM physicians were known to students and became their admired seniors. It greatly increased the affection component of attitude, as shown in the result of question 11.

### The Effect of TCM Course on Behavioral Tendency Component of Attitude in FYP

The behavioral tendency component of attitude also improved after the TCM course among FYP students. The clinical internship after the theoretical lecture was also conducted to change the behavioral component of attitude. The internship aimed at integrating TCM theory with practice. In this way, abstract TCM theories and methods would be specified [25]. For example, students visited patients with different syndromes, and they were taught the yin and yang attributes of syndromes according to the Yin-Yang theory. The internship also showed the students a complete diagnosis and treatment process of TCM, which included 4 diagnostic methods (ie, observation, auscultation and olfaction, inquiry, and pulse feeling and palpation), syndrome differentiation, and treatment as well [26]. During the internship, students experienced some unique diagnostic methods of TCM, such as tongue diagnosis, pulse feeling, and palpation. Students even tried the needling sensation (Deqi) themselves in the internship. Hence, the clinical internship afforded the students a hands-on and introspective encounter with TCM, rather than mere hearsay and boastful claims. It was of great importance for students to learn about the unique advantages of TCM, to eliminate prejudices about TCM, and, finally, to make a more objective and comprehensive judgment about TCM.

### The Effect of Chinese Medicine Celebrity and TCM Course on Attitude in FYP

There was also an interesting result that before the course, students who admired Chinese medicine celebrity worship had a more positive attitude toward TCM compared with those who did not. However, after the course, there was no intergroup difference in attitudes between these 2 groups of students, although there were intragroup differences. In other words, the course made the attitudes of the 2 groups of students, which were originally different, become consistent and significantly more positive. The hierarchical effect may explain this interesting result. Students who did not admire Chinese medicine celebrities may follow a standard learning hierarchy. In this learning hierarchy, cognition was the foundation, which in turn affects emotions and ultimately behavioral tendencies. Students who admired Chinese medicine celebrities followed an experiential hierarchy, in which emotions were the foundation that influenced behavior and ultimately cognition.

### Why the TCM Course Did Not Affect Cognitive and Affective Components of Attitude in EYP

Compared with FYP students, it was very interesting that TCM courses did not significantly change the total score or the scores in the cognition and affection domains in EYP students. Some facts attracted our attention that might be reasons for quite different results. First, 5th-grade EYP students had completed WM diagnostics and accessed almost all the clinical professional courses, including internal medicine, surgery, gynecology, and pediatrics, alike. Moreover, they had determined their future professional major and selected their doctoral supervisors. Therefore, long-term WM clinical training had already established an almost complete WM knowledge system and the mode of thinking for students in EYP. It was difficult to be changed by TCM learning at a relatively later stage. It was a formidable challenge for EYP students to acknowledge and assimilate TCM, given their preconceived learning of WM, because of the fact that these 2 domains are founded on divergent theories and modalities [27-29]. However, for FYP students, 3-year learning in basic and clinical bridging courses has not yet fully westernized their thinking. They were relatively simple to change through TCM learning. This might be one of the critical reasons why total scores and scores in cognition and affection did not increase in EYP. The findings of this study were in accordance with the report of Hon et al [8]that preclinical students were more positive toward TCM compared to students in clinical years, the latter showing a more negative attitude toward TCM after studying WM. In addition, the small sample size included (36 EYP students) might be another important reason for the absence of a difference between before and after tests. It is difficult to avoid the fact that the college entrance examination enrollment of the EYP is very limited. Future studies should adopt more rigorous experimental designs to address this limitation, for example, a combination of effects of several consecutive years should be feasible.

### The Effect of TCM Course on Behavioral Tendency Component of Attitude in EYP

The behavioral tendency component of attitude among EYP students was improved after the course. Behavioral intention is different from actual behavior, but it is an individual’s behavioral readiness toward a certain object, representing a possible behavioral tendency. The result suggests that after learning TCM, EYP students gained more in-depth exposure to another medical strategy. It is greatly different from WM yet effective, well founded, and uniquely advantageous, and can solve some problems that WM cannot solve. Therefore, they may be willing to use TCM for health maintenance, to use the knowledge of TCM to safeguard their parents’ health, to learn more about TCM, and to consider using TCM to solve patients’ illnesses in future clinical work.

### The Effect of TCM Course on Selection Preference of Different Therapeutic Strategies in FYP

The purpose of this part of the questionnaire was to test the behavior tendency in specific clinical conditions. The test was designed to simulate a physician’s daily work, such as making an appropriate treatment plan for different types of diseases or, at least, giving proper referrals for different patients. After completing the TCM course, more FYP students realized that TCM and WM were very effective in treating acute diseases, which broke the stereotype that TCM was only suitable for chronic diseases. Many real cases they witnessed during their TCM internship may have influenced their selection preference. For example, a TCM physician cured a child with unexplained persistent high fever with a combination of xiaochaihu decoction and gypsum soup. As for chronic diseases, more FYP students tended to choose TCM treatment after the course. These diseases often have a long course and complex condition, requiring long-term management and treatment. The TCM course taught students that TCM relies on its unique theoretical system and rich practical experience and has demonstrated significant advantages and potential [30]. It emphasizes a holistic concept and treatment with syndrome differentiation for chronic diseases, which helps physicians treat patients using the overall situation, comprehensively considering the patients’ physical condition, lifestyle habits, emotional factors, and other aspects. As for organic diseases, more FYP students realized that the strategy of using both TCM and WM is better. This result is reasonable and explainable. It is widely recognized that WM is the mainstream and effective treatment for organic diseases. However, during the internship, students found that participation of TCM in the treatment of organic diseases accelerated recovery, minimized side effects, and increased patient comfort. Thus, they tended to use TCM and WM for organic diseases.

### The Effect of TCM Course on Selection Preference of Different Therapeutic Strategies in EYP

After the TCM course, more EYP students chose TCM and WM to treat internal diseases. EYP students had completed many clinical courses, so they were more likely to divide diseases into internal and surgical diseases according to their scope and treatment methods. Generally speaking, internal medicine diseases can be treated with medication. By learning the basic theory of TCM, along with Chinese pharmacy and Chinese medical formulas, they understood how TCM cures diseases. It is greatly different from that of WM but is a good supplement and promotion to WM. However, for surgical diseases, surgical treatment remains the first choice undoubtedly. Nevertheless, EYP students found that the TCM therapies, such as acupuncture and massage, help restore gastrointestinal, cognitive, and limb functions in postoperative patients. Therefore, they preferred to use a combination of TCM and WM to address perioperative disorders. Mental illness is another major category of disease. The therapeutic effect of antipsychotic drugs is limited, and there are many side effects. Mental illness belongs to the category of emotional disorders in TCM. By learning TCM etiology and pathogenesis, students understood that emotional disorders are related to various factors, such as organ dysfunction, yin-yang imbalance, and so on. Common treatment methods include regulating qi and relieving depression, calming the heart and eliminating phlegm, purging the liver and clearing fire, and so on. EYP students also found that TCM and WM treatment showed a satisfactory effect in patients with mental illness. Thus, more EYP students preferred a TCM and WM strategy for mental illness. The results additionally aligned with a previous study, which found that TCM was not practiced in isolation but in conjunction with WM [17]. The increased participation of TCM in treatment was expected to enhance its effectiveness.

### Implications of Findings

The findings of the present study highlight the importance of integrating TCM into WM education and practice. It will lead to more holistic and patient-centered care, enhance clinical outcomes, and contribute to the development of integrated health care policies. Future research should continue to explore the long-term effects of TCM education on WM students’ clinical practice and the potential benefits of integrating TCM and WM in various health care settings.

### Comparison With Prior Literature

The findings of this study align with and extend several key themes observed in the existing literature on the integration of TCM and WM, particularly in the context of medical education and attitude formation. This study builds on existing literature by confirming the positive impact of TCM education on WM students’ attitudes and by extending the understanding of how this impact varies by educational stage and specific disease contexts. The detailed analysis of attitude components and the introduction of novel factors, such as celebrity worship, provide new insights that can inform future research and educational practices in the integration of TCM and WM.

### Strengths and Limitations

This study has several distinct advantages that contribute to its overall quality and relevance. The study used a self-controlled design, which is a robust method for assessing changes in attitudes over time. By comparing the same group of students before and after the TCM course, the study effectively controls for individual differences and external variables, providing a more accurate assessment of the impact of the TCM course. The study used the ASTCM, a validated and reliable tool designed specifically to measure medical students’ attitudes toward TCM through a multidimensional analysis. This ensured that the measurements are accurate, reproducible, and reflective of the true changes in students’ attitudes. Some inevitable limitations in this study should be pointed out. First, a self-administered questionnaire was used instead of an interview. The reliability of the answers may be discounted. The qualitative interview should be included to make the study more persuasive in our future work. Second, the questionnaire is a kind of survey based on text reading and filling in answers. To avoid the feeling of boredom in participants, the questionnaire was designed to be relatively brief. Thus, it was impossible to have an in-depth examination. Moreover, it is an online questionnaire. Researchers possess a limited understanding of the actual identity of the participant, the mood of the participant at that time, and whether the process of finishing the questionnaire was influenced by others. Finally, the sample size of EYP students was relatively small. In the 2-tailed *t* test, the smaller sample size makes it harder to gain differential results. Thus, the results may be influenced. A sufficient sample size or combination of effects of EYP for several consecutive years is expected in the future.

### Conclusions

The study showed that offering a TCM course increased a positive attitude toward TCM in students majoring in WM. The results will provide recommendations with regard to the TCM curriculum arrangement in institutions of WM learning.

## Supplementary material

10.2196/55972 Multimedia Appendix 1 Ethical approval document.

## Abbreviations

ASTCM: Attitude Scale of Traditional Chinese Medicine
EYP: 8-year program
FYP: 5-year program
TCM: traditional Chinese medicine
WM: Western medicine
