# Supplementary material for: The Effect of a Traditional Chinese Medicine Course on Western Medicine Students’ Attitudes Toward Traditional Chinese Medicine: Self-Controlled Pre-Post Questionnaire Study
Source: JMIR Med Educ. 2026 Jan 16;12:e55972. doi: 10.2196/55972 (PMC12810946; doi:10.2196/55972)
Supplement: Multimedia Appendix 1 [file mededu-v12-e55972-s001.pdf]

湖南第一师范学院伦理审查审批件

该项目的研究过程经二级学院初审、学校科技伦理委员会审核，符合伦理原则，同意申请。

项目名称	中医必修课程对西医学生中医态度的影响研究		
经费来源	自筹	申请日期	2022 年 2 月 3 日
项目负责人	何浩宇	职称/学位	讲师
院系（部门）	教育学院	联系方式	15211040421
审查日期	2022 年 3 月 3 日	审查地点	湖南第一师范学院
批准文件	见附件		
伦理编号	20（22）伦理（02）号		
联系人	张艳	联系电话	0731-88227250
是否涉及到实验仪器	☑是 □否	签章<br>（设施负责人）	
伦理委员会<br>湖南第一师范学院 科技伦理委员会		签章	

湖南第一师范学院科技伦理审查申请表

编号：2022伦理02号

（适用于涉及人的生物医学研究）

项目名称	中医必修课程对西医学生中医态度的影响研究				
项目负责人	何浩宇	职称	讲师	从事专业	心理学与心理健康
所在学院	教育学院			电话/邮箱	15211040421<br>hehaoyu@hnfnu.edu.cn
项目来源	无			研究期限	6个月
审查类型	初审☑　　复审☐　　延长☐				
审查文件	1.科技伦理审查申请表　　2.研究人员信息<br>3.研究方案及相关材料　　4.知情同意书　　5.其他				

申请人（项目负责人）承诺：

以上内容（包括提交的文件）均属实，如获批准，我将严格按照提供的方案进行研究，并遵守湖南第一师范学院科技伦理委员会的相关规定。

申请人（项目负责人）签字：何浩宇　　日期：2022年2月3日

审议内容	审议意见	
1.研究者资格、经验是否符合要求	☑符合	☐不符合
2.研究方案是否符合科学性和伦理原则	☑符合	☐不符合
3.受试者的入选和排除标准是合适或公平	☑是	☐否
4.受试者所承担风险的程度	☑小	☐大
5.知情同意书的信息是否完整、易懂	☑完整、易懂	☐不完整
6.对受试者的资料是否采取了保密措施	☑有	☐无
7.研究对象是否为弱势群体	☐是	☑否

8. 弱势群体受试者可能遭受的风险程度有无增加	□有	☑无
9. 研究人员与受试者之间有无利益冲突	□有	☑无

二级学院审查意见：

同意

二级学院负责人签字（盖章）：[illegible]

湖南第一师范学院 教育学院

2022 年 2 月 5 日

审查意见：

☑同意进行研究　□作必要的修正后同意进行研究　□作必要的修正后重审

□不同意研究　□暂停或终止先前已批准的研究

说明理由：符合伦理审查要求

主任（签章）：

学校科技伦理委员会（盖章）

湖南第一师范学院 科技伦理委员会

2022 年 3 月 3 日

注：本表打印一式三份，电子稿发送至 kyczy@hnfnu.edu.cn，按表格提示完成签字盖章流程，完成后科研处备份 1 份。表格中的编号由科研处填写。
